# 338. Evaluating the Clinical Impact of Molecular Drug Susceptibility Testing in Patients with Drug-Resistant Tuberculosis in Los Angeles County

**DOI:** 10.1093/ofid/ofaf695.121

**Published:** 2026-01-11

**Authors:** Kyla Sherwood, Shom Dasgupta-Tsinikas, David L Fuller

**Affiliations:** Palo Alto VA , Los Angeles, CA; Los Angeles County Department of Public Health, Los Angeles, California; Los Angeles County Department of Public Health, Los Angeles, California

## Abstract

**Background:**

Prompt identification of drug-resistant tuberculosis (DR-TB) is crucial for the timely initiation of effective therapy, but phenotypic drug susceptibility testing (pDST) takes weeks to provide results. Advanced molecular drug susceptibility testing (mDST) allows for rapid detection of mutations conferring drug resistance. This retrospective, cross-sectional study evaluated the association of mDST with clinical outcomes in patients with DR-TB in Los Angeles County from 2018-2022.

**Table 1.**
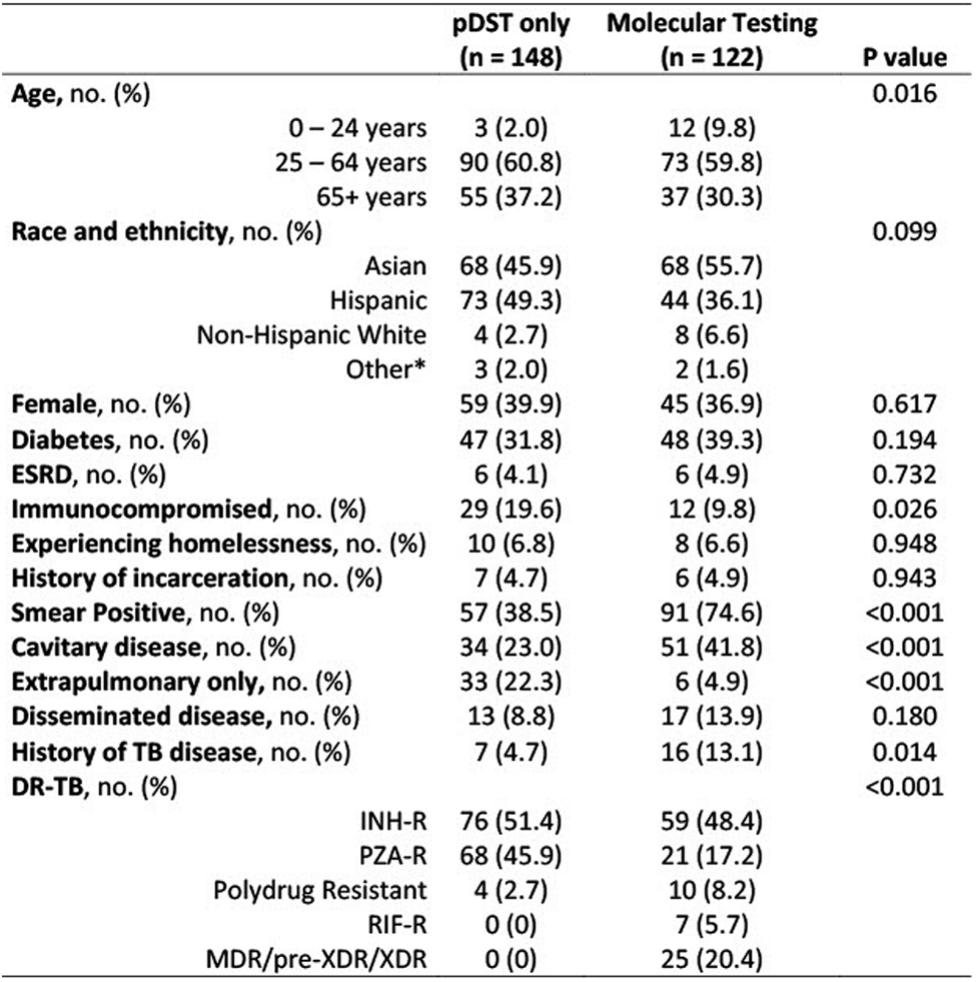
Demographic and clinical characteristics of the study population *Other includes Non-Hispanic Black, Native Hawaiian, and Other Pacific Islander. Abbreviations: ESRD = End-stage Renal Disease; TB = Tuberculosis; DR-TB = Drug-resistant Tuberculosis; INH-R = Isoniazid-resistant; PZA-R = Pyrazinamide-resistant; RIF-R = Rifampin-resistant; MDR = Multidrug-resistant; XDR = Extensively drug-resistant

**Table 2.**
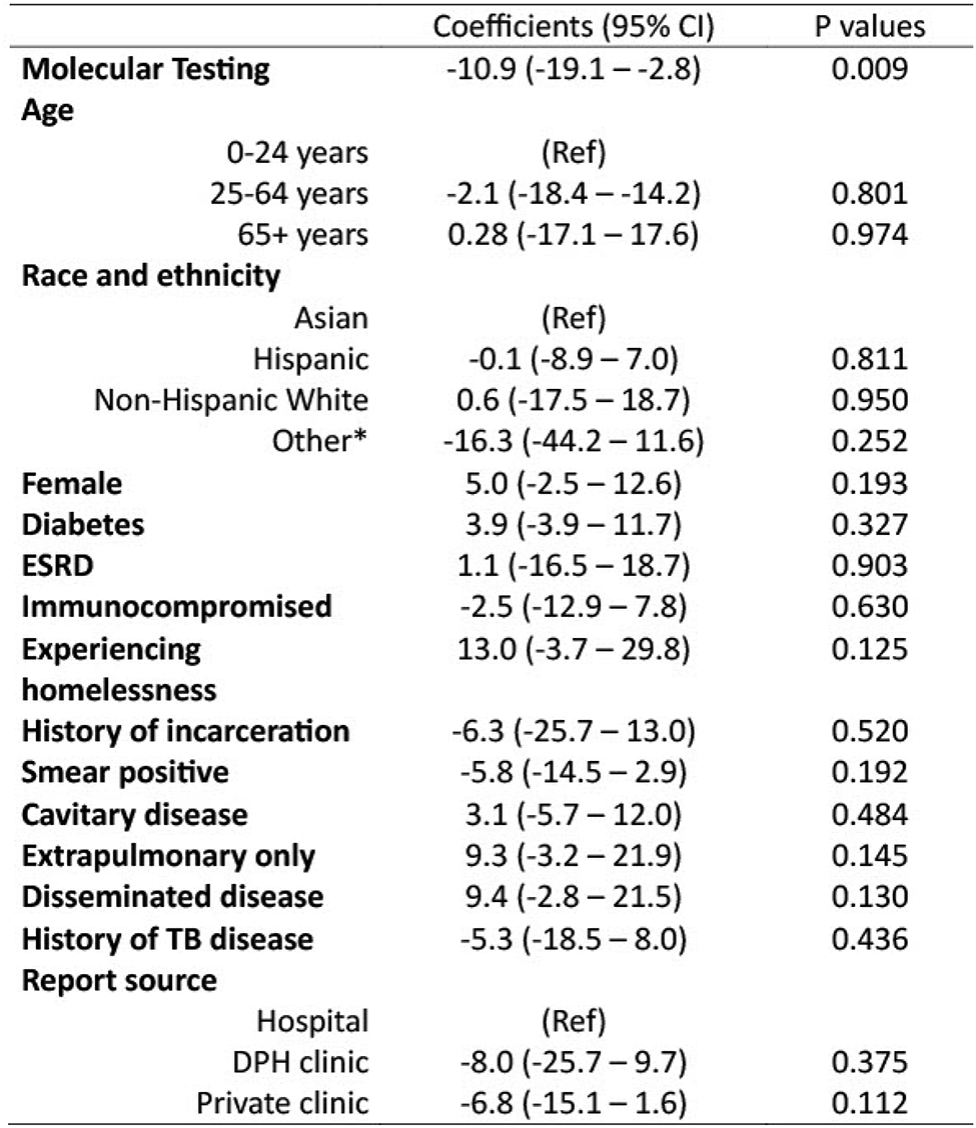
Multivariable linear regression coefficients for drug-resistant tuberculosis patients receiving pDST only as compared to mDST The units of the coefficients are in days. *Other includes Non-Hispanic Black, Native Hawaiian, and Other Pacific Islander. Abbreviations: ESRD = End-stage Renal Disease; TB = Tuberculosis; DPH = Department of Public Health.

**Methods:**

The study population was divided into two groups: pDST only (excluding GeneXpert rifampin resistance testing) vs. use of mDST (pyrosequencing [PSQ] or molecular detection of drug resistance testing [MDDR]). Covariates included demographics, medical and social history, and features of TB disease. The primary outcome was time to lab-informed treatment for DR-TB, defined as first use of a TB regimen in response to DST results. Secondary outcomes were time until sputum culture conversion, adverse drug events, and total treatment duration. Multivariable linear regression was performed to evaluate outcomes associated with the use of mDST.

**Results:**

Of 270 patients with DR-TB, 148 (55%) had pDST only and 122 (45%) had mDST. In the mDST group, PSQ first identified resistance in 70 (57%) patients, MDDR in 7 (6%), and pDST in 45 (36%). The use of mDST was associated with a mean 11-day reduction in time to lab-informed treatment compared to only pDST (P < 0.05). No significant differences in secondary outcomes were noted between groups. Further evaluation of mDST modalities revealed PSQ use was associated with a mean 17-day reduction in time to lab-informed treatment (P < 0.05), but no significant difference was associated with MDDR use.

**Conclusion:**

In patients with DR-TB, the use of mDST was associated with shorter time to lab-informed treatment, primarily driven by PSQ testing, likely due to PSQ’s rapid turnaround time. Notably, only 45% of DR-TB patients received mDST, underscoring the importance of greater access to mDST which may improve clinical outcomes. Better prediction tools to identify patients with DR-TB and streamlined testing protocols should be developed to best facilitate mDST.

**Disclosures:**

All Authors: No reported disclosures

